# RefGenes: identification of reliable and condition specific reference genes for RT-qPCR data normalization

**DOI:** 10.1186/1471-2164-12-156

**Published:** 2011-03-21

**Authors:** Tomas Hruz, Markus Wyss, Mylene Docquier, Michael W Pfaffl, Sabine Masanetz, Lorenzo Borghi, Phebe Verbrugghe, Luba Kalaydjieva, Stefan Bleuler, Oliver Laule, Patrick Descombes, Wilhelm Gruissem, Philip Zimmermann

**Affiliations:** 1 Department of Biology, ETH Zurich, 8092 Zurich, Switzerland; 2 Institute of Theoretical Computer Science, ETH Zurich, 8092 Zurich, Switzerland; 3 Genomics Platform, NCCR 'Frontiers in Genetics', University of Geneva, 1211 Geneva 4, Switzerland; 4 Physiology Weihenstephan, TUM, Weihenstephaner Berg 3, 85354 Freising, Germany; 5 NEBION AG, Hohlstrasse 515, 8048 Zurich, Switzerland; 6 Western Australian Institute for Medical Research, UWA Centre for Medical Research, Perth, Australia

## Abstract

**Background:**

RT-qPCR is a sensitive and increasingly used method for gene expression quantification. To normalize RT-qPCR measurements between samples, most laboratories use endogenous reference genes as internal controls. There is increasing evidence, however, that the expression of commonly used reference genes can vary significantly in certain contexts.

**Results:**

Using the Genevestigator database of normalized and well-annotated microarray experiments, we describe the expression stability characteristics of the transciptomes of several organisms. The results show that a) no genes are universally stable, b) most commonly used reference genes yield very high transcript abundances as compared to the entire transcriptome, and c) for each biological context a subset of stable genes exists that has smaller variance than commonly used reference genes or genes that were selected for their stability across all conditions.

**Conclusion:**

We therefore propose the normalization of RT-qPCR data using reference genes that are specifically chosen for the conditions under study. RefGenes is a community tool developed for that purpose. Validation RT-qPCR experiments across several organisms showed that the candidates proposed by RefGenes generally outperformed commonly used reference genes. RefGenes is available within Genevestigator at http://www.genevestigator.com.

## Background

### Rationale for using reference genes

Reference genes, sometimes also called "housekeeping genes", frequently serve as internal controls in transcript quantification assays such as RT-qPCR. The need for internal controls in such assays arises from sample to sample biases related to variability in total RNA content, RNA stability, enzymatic efficiencies, or sample loading variation. To correct for this, the expression levels measured are frequently normalized to internal control genes. Ideally, such genes are expected to be invariable in their expression and therefore correlate strongly with the total amounts of mRNA present in each sample. Commonly used reference genes, such as beta-actin (ACTB), ubiquitin (UBQ), the 18 S ribosome small subunit (18S), beta-glucuronidase (GUS), or glyceraldehyde 3-phosphate dehydrogenase (GAPDH), have a strong tradition and historical track record. In fact, many manufacturers provide "housekeeping gene panels" containing a dozen such genes thought to be generally stable based on their biological function. In many laboratories, they are used as "general purpose" reference genes for a wide variety of experimental conditions.

### Problems associated with reference genes

Despite their wide-spread use, the suitability of reference genes for any type of experiment is not given *a priori*. In fact, two types of problems can occur: 1) their expression can vary considerably depending on the experimental condition being tested, and 2) the majority of these genes is very strongly expressed, often resulting in a discrepancy in transcript abundance of several orders of magnitude relative to the target gene transcripts being quantified. Both sources of error can cause significant biases that can ultimately lead to wrong data interpretation, especially in those cases where a single gene is used for normalization. For example, [1-5] have described various problems associated with commonly used reference genes.

### Current approaches for improved data normalization

Although limitations are universally recognized, still many laboratories use reference genes without appropriate validation [6,7]. In an effort to improve the quality and normalization of RT-qPCR data, several approaches have been proposed.

A first approach consists of validating reference genes using data obtained from RT-qPCR data. Frequently, several genes are evaluated in parallel and the most stable are selected for further experimentation. So far, most studies have focused on validating a subset of commonly used reference genes for specific contexts such as tissue types. Overall, it appears that no reference gene was generally suitable for any type of context, and that the best candidates differ between different tissues. In some cases, even opposite results were found for different tissues. For example, Meller et al. [3] analyzed seven commonly used reference genes for their expression level stability in placenta and reported that TBP and SHDA exhibited highest stability. In contrast, of the 10 commonly used reference genes tested by Zhang and colleagues in human neutrophils [5], TBP appeared to be the least stable. A list of similar studies in which validations were performed in a variety of organisms and tissues is available in Additional file 1. Although these studies have their merits, they try to identify the best candidates from a small and *a priori *set of genes, assuming that at least one or a few of them are suitable for the experimental context under study.

A second approach is to normalize against multiple reference genes and to use appropriate statistical models to improve the selection of genes with minimal variance [8-14]. Most current software packages for RT-qPCR data analysis have incorporated one or the other of these methods. Three of the most popular algorithms are GeNorm [13], Norm finder [8] and Bestkeeper [15].

A more recent, data-driven method consists of using quantile normalization rather than reference genes, but this approach is designed for high-throughput RT-qPCR experiments involving many genes. For studies involving one or a few genes, data normalization using internal control genes remains the method of choice, provided a proper choice of reference genes and normalization algorithms [16,17].

A fourth and quite successful approach has been to search for reference genes from a genome-wide background using microarray data. In most cases, large sets of microarray data were compiled for a specific or for a subset of conditions, and stable genes identified within these datasets were validated and recommended for future use. Validation experiments generally showed that these genes performed better than commonly used reference genes. For example, Czechowski and colleagues [18] selected stably expressed genes for a variety of experimental series for Arabidopsis. Partial overlap was found between some of these conditions, but overall each series had its specific set of most stably expressed genes. Saviozzi et al. [19] performed a meta-analysis of lung cancer transcription profiles and validated several new reference genes for this particular context. Other similar studies were done e.g. for T-helper cells [20], adipose tissues [21], peripheral blood [22], various human samples and cell lines [23], breast tumor tissues [24], breast cancer [25], human myocardium [26], mouse (universal) [27], and human (universal) [28]. The use of microarrays to identify candidate reference genes for RT-qPCR normalization has been successful, but this extrapolation requires some precautions due to differences in the choice probe sequences between the two technologies (e.g. Affymetrixprobes typically target the 3' region of a transcript). Additionally, in microarray data, multiple probes (or probe sets) targeting the same transcript may exhibit different stability values due to cross- or weak hybridization. Therefore, in a RT-qPCR assay, novel candidates should always be validated against reference genes previously used in the laboratory.

### Conclusions from published data

From the experimental evidence accumulated and published so far, we conclude that there are probably no genes that have a sufficient overall expression stability to be suitable for any type of assay. As previously suggested, reference genes should be selected according to the nature of the study [6,7], for example according to the tissue type or stage of development, and should ideally not be sensitive to perturbations such as external stimuli, diseases, or even to genetic modifications. Moreover, reference genes are preferably selected from the complete genome rather than from a handful of commonly used reference genes.

### Hypotheses

In this study, we have examined how to find the best possible candidate reference genes for specific experimental contexts, starting from a genome-wide set of genes. To do so, we defined an "ideal reference gene" as a gene which 1) has the most stable transcript abundance within the biological context of a specific experiment, and 2) has an abundance of transcripts similar to that of the target genes under investigation. The hypotheses that we tested were the following:

1. No genes are generally stably expressed; all genes are regulated to a certain extent (non-generality clause)

2. For each biological context there exists a subset of genes with smaller expression variance in this context than genes that are most stably expressed across many conditions (context-specificity clause)

3. Genes that are stably expressed in a given biological context are likely to be stably expressed in similar contexts (context- relatedness clause)

4. Genes that are stably expressed in a given tissue of an organism are likely to be stably expressed in the same tissue from closely related species (orthology clause)

In this paper, we tested and substantiated these hypotheses by using data from more than 40,000 quality controlled and manually annotated microarrays from a wide variety of experimental contexts and from several organisms. We studied the properties of the expression level of genes across various microarray types. Finally, to validate our approach, we identified novel reference genes, examined their individual properties, and compared their performance to commonly used reference genes using RT-qPCR assays. We also present an online tool which helps to identify genes that show high expression stability in a chosen set of conditions. Researchers can thereby identify, from all genes represented on the microarrays, those which are most stably expressed across conditions that are similar to that of their own experiments, providing them with an objective choice of candidate reference genes.

## Results

### Datasets used in this study

The Genevestigator database contains a large set of systematically annotated and quality controlled microarray data from several organisms [29]. Owing to the high reproducibility of the Affymetrix system, its streamlined labeling and hybridization protocols, the normalization methods used, as well as our quality control measures, expression data from different laboratories show a high degree of homogeneity. The database therefore offers a unique opportunity to search for genes that have particular expression characteristics across experiments, for example reference genes that have minimal variance across a chosen set of conditions.

### Validating our hypotheses

#### Hypothesis 1 (non-generality clause)

**Figure 1:**
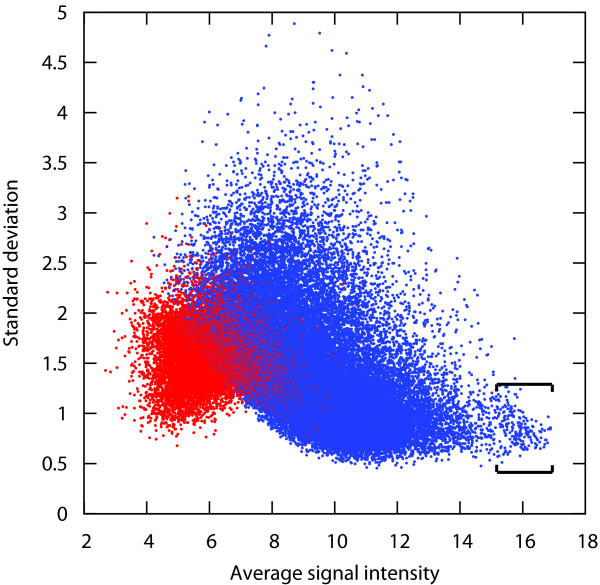
**Variance of gene expression relative to transcript abundance**. Standard deviation versus average transcript abundance of approximately 47,000 probe sets across 5014 AffymetrixHuman133 2.0 arrays. Spots in blue show probe sets with Present calls in at least ten percent of arrays, while spots in red are those with Absent calls in more than ninety percent of arrays. The probe sets contained between the square brackets are highly enriched in ribosomal protein genes, but include many of the commonly used reference genes (e.g. GAPDH, ACTB, B2 M, PPIA, EIF1, ACTG1, UBC, EEF1G, TUBA1B, EEF1A1, TPT1).

Public experimental evidence accumulated and published so far seems to suggest that there are no genes whose expression is universally stable across any type of condition. To verify this hypothesis, we measured the standard deviation of gene expression across large sets of Affymetrix arrays from various array types and covering a broad variety of conditions. This analysis was carried out for human, mouse and Arabidopsis. The results show that for all three organisms tested, the ranges of standard deviation of gene expression across the complete available datasets were approximately 15-fold, with values mostly varying between 0.5 and 5 (Figure 1; see also Additional file 2). Commonly used reference genes were generally located within the range of SD between 0.5 and 1.0. However, a large portion of the genes have SD values in this range. For instance, for the human data set shown in Figure 1, more than 8000 other probe sets were located within this range of SD. It is unlikely that the expression of one fifth of the transcriptome is sufficiently invariant so that any of them could be used for normalization. Furthermore, no genes were found to have a standard deviation distinctly lower than the bulk of the remaining genes and could be declared as "universally suitable reference genes". Genes with a high average expression level showed slightly lower variance of expression across these datasets. This effect could be due to the normalization method used in this study (MAS5/GCOS) or to saturation effects. Nevertheless, it is interesting to note that most reference gene panels tend to choose very highly expressed genes. In Figure 1, a partly distinct cloud of probe sets was formed in the very high range of expression and low range of standard deviation. This cloud is enriched in cross-hybridizing probe sets, mainly probe sets hybridizing transcripts from the same family of genes. The vast majority of them represent genes encoding ribosomal proteins, while from the remaining genes from this cloud several commonly used reference genes were identified, such as GAPDH (glyceraldehyde-3-phosphate dehydrogenase), ACTB (beta-actin), UBB (ubiq-uitin B), B2 M (beta-2-microglobulin), PPIA (pep-tidylprolyl isomerase A (cyclophilin A)), EIF1 (eukaryotic translation initiation factor 1), TUBA1B (tubulin, alpha 1b), HSP90AA1 (heat shock protein 90 kDa alpha (cytosolic), class A member 1), UBC (ubiquitin C), H3F3A (H3 histone, family 3A) and EEF1G (Eukaryotic translation elongation factor 1 gamma). Similar observations were obtained by analyzing data from various array types and organisms, including human, mouse and Arabidopsis (see Additional file 2). A further piece of evidence in support of Hypothesis 1 was that from the top 50 transcripts that were most stable across all conditions in Figure 1, all of them were found to have a considerable variability of expression in at least five distinct tissue types from a set of 186 tissues available in Genevestigator (see Additional file 3). For one third of these tissues (60) the standard deviation of expression was very high for at least one of these 50 "generally stable" genes, indicating that this effect is not due to a common set of biased experiments. These genes would clearly not be suitable to normalize data obtained from these particular experimental conditions, even if their overall expression stability is high.

#### Hypothesis 2 (Context-specificity clause)

**Figure 2:**
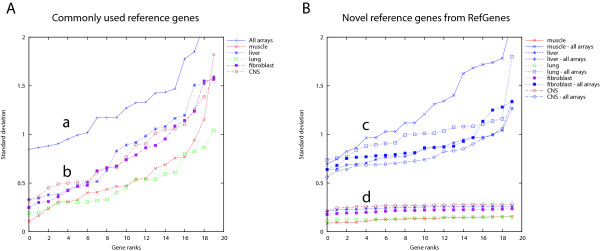
**Standard deviation of commonly used and novel reference genes**. Standard deviation (SD) of gene expression of commonly used reference genes (A) across all samples (a) or across subsets of tissue-specific samples (b) from the AffymetrixMouse430 2.0 array dataset. In (B), for each subset of tissue-specific samples, the most stable genes were identified using Genevestigator RefGenes. Their respective expression SD across all arrays (c) or across subsets of tissue-specific arrays (d) is shown. The control reference genes used in this study and shown in plot A were: HSP90AB1, TFRC, B2 M, NONO, GUSB, UBC, ACTB, H2AFZ, POLR2A, TUBB4, HIST2H2AA1, RPL22, GAPDH, YWHAZ, CANX, CYC1, SDHA, EIF4A2, ATP5B, and EEF1E1.

Our second hypothesis was that for each biological context, a distinct set of genes exists with lower variance within this context than genes selected for their stability over a variety of different contexts. To verify this hypothesis, we created, from a compendium of 3051 mouse arrays (Mouse430 2.0) from Genevestigator, selections of arrays representing various tissue types (muscle, liver, lung, fibroblast, Central Nervous System). As control we used the complete set of 3051 arrays covering a wide variety of contexts. We chose to work with the mouse dataset because it contained several tissue types with high data coverage. For each of these array selections, we calculated the standard deviation (SD) for each probe set available on the array and ranked them from lowest to highest SD. Figure 2A shows the results for 20 commonly used reference genes across all arrays (a), and across tissue-specific subsets of arrays (b). In Figure 2B, for each tissue type we identified the top-20 genes with lowest SD and ranked them by increasing SD (d), and as a control, we show their respective ranked SD across all arrays (c). Two observations can be made:

1) Genes selected for their stability within a chosen tissue type had a lower SD of expression than commonly used reference genes, both within these tissue types (up to 4-fold lower) and also as measured across all arrays (up to 1.5 fold lower).

2) For each tissue, the range of SD of the top 20 most stable transcripts was within 1.5 fold difference between the most stable and least stable gene (see also Additional file 4). In contrast, the SD of the 20 commonly used reference genes varied more than 5-fold, irrespective of the tissue type, indicating that for each tissue type several of these genes would be unsuitable for data normalization. None of the 20 commonly used reference genes was systematically ranked within the top 5 genes across every tissue type, and some even had highly variable ranks. For example, TFRC (probe set 1452661_at) had rank 1 in spinal cord and rank 20 in liver (see Additional file 5).

**Figure 3:**
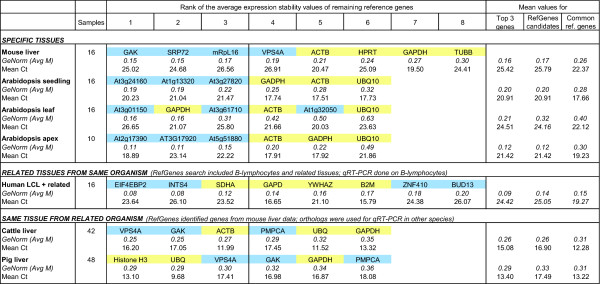
**RT-qPCR validation experiments**. GeNorm and mean Cq values from the RT-qPCR validation experiments carried out on samples from Mouse, Arabidopsis, Human, Cattle, and Pig. Novel reference gene candidates (blue) identified with the RefGenes tool were compared to commonly used reference genes (yellow). The first section shows the results for tissues that were abundantly represented in Genevestigator and for which novel reference genes were proposed by RefGenes. In this case, the candidates proposed by RefGenes generally performed better according to GeNorm than commonly used reference genes. The second section shows RT-qPCR results for human lymphoblastoid cell lines (LCLs). Novel reference genes were identified from a a set of human LCLs and related tissues, because there were too few arrays available for this specific tissue type alone. The third section shows results in cattle and pig liver. Because at the time of writing this article no cattle and pig expression data were available in Genevestigator, novel reference gene candidates were identified with RefGenes from mouse liver samples and extrapolated to orthologs from cattle and pig. The GeNorm values indicated ("GeNorm (Avg M)") represent the average of the expression stability values M of the remaining genes after removal of the least stable one (see full results in Additional file 6). For all experiments, the average GeNorm and Cp values for the top 3 genes, the novel genes identified from RefGenes, and the commonly used reference genes are shown. In all cases, the average Cp values of candidates found by RefGenes were lower than those of commonly used reference genes.

To substantiate these findings, we carried out two independent RT-qPCR experiments with tissues from mouse and Arabidopsis samples. For each experiment, we used the RefGenes tool from Genevestigator (see below) to find candidate reference genes for specific tissue types, and then tested these candidates against commonly used reference genes using GeNorm. The first experiment was carried out with mouse liver. The stability of four control reference genes (GAPDH, TUBB, ACTB, and HPRT) was compared to that of four novel reference genes (vps4a, srp72, mRpL16, and GAK) identified as being highly stable across a set of 197 Affymetrix arrays profiling mouse liver samples from 7 distinct public experiments available in Genevestigator. For each gene, measurements were done in triplicate for 16 liver samples, and all reactions were run simultaneously. From these eight genes, GeNorm iteratively removed the least stable ones in the following order: TUBB, GAPDH, HPRT, ACTB, VPS4A, mRpL16, with srp72 and GAK being the two most stable genes (see Figure 3). In almost every iteration GAK appeared to be the most stable gene (see Additional file 6). This experiment proved that liver-specific stable genes, as identified from Affymetrix microarray data from liver samples, outperformed commonly used reference genes for the normalization of RT-qPCR data from liver.

The second experiment consisted of identifying genes that are stable in seedlings, leaves and shoot apex of the model plant Arabidopsis, and to compare their expression with that of reference genes commonly used in this species using RT-qPCR. For each tissue type, 16, 16, and 10 samples were used, respectively. The results are provided in Figure 3. For seedlings and shoot apex, all candidates proposed by RefGenes showed higher stability in this experiment than the reference genes GAPDH, ACTB and UBQ10. In leaves, the most stable genes were GAPDH and one of the novel genes identified by RefGenes (same score). Overall, the RefGenes candidates had ranks 1, 3 and 5. In the RT-qPCR experiment, GAPDH performed better than one would have expected from the microarray data, in which the novel candidates were found to be more stable. This illustrates potential differences that may occur due to the different size and composition of experiments and samples underlying each of these datasets. In fact, the microarray dataset selected was composed of a large number of leaf samples from a variety of experimental conditions, whereas in the RT-qPCR assay there were 16 samples grown in the same conditions. It is also possible that there are discrepancies between the two technologies, e.g. due to the targeting of different regions or splice variants.

Overall, the results from mouse and Arabidopsis substantiate this hypothesis. The tissue-specific selection of reference genes using microarray data carried out in similar conditions allows to identify novel genes having higher expression stability and a more suitable expression range than commonly used reference genes. For both organisms and across all genes tested, the Cq values (i.e. the number of PCR cycles that elapse before a given threshold concentration of PCR product is reached) from the novel RefGenes candidates were higher than those of commonly used reference genes and closer to Cq values commonly found for most genes from the genome (see Additional file 7 for original experimental data).

#### Hypothesis 3 (Context-relatedness clause)

**Figure 4:**
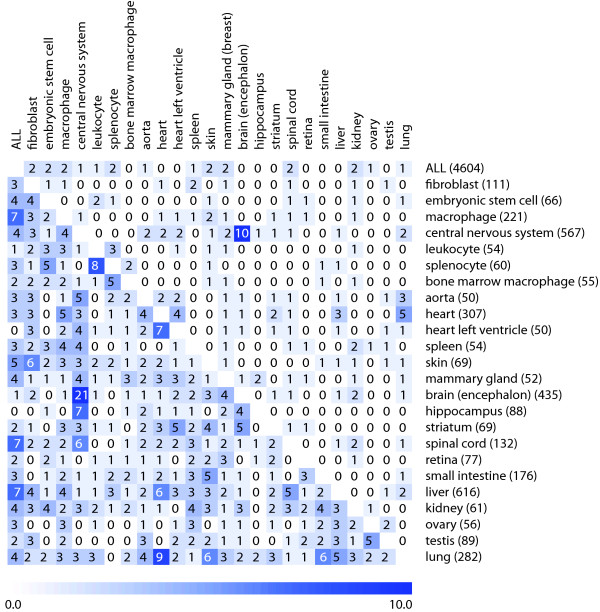
**Overlap of most stable probe sets between tissue types**. Overlap of the top 20 (top right section) and top 50 (lower left section) candidate reference genes identified by RefGenes in different tissue types. The number of samples available for each category is indicated in parenthesis.

Our third hypothesis was that related tissue types have overlapping sets of genes that are most stable within these tissues. To verify this hypothesis, we selected 24 individual tissue types for which at least 50 arrays from 3 or more independent experiments were available in Genevestigator. We then compared the overlap of the top 20 and top 50 genes that were most stable in each of them and, as an additional comparison, across a selection of all tissues (Figure 4). The results show that in the top 20 comparisons, very few genes overlapped between any pair of the tissue types, except for central nervous system (CNS) versus brain. This is a particular case, as the selection of encephalon samples is contained as a subset of the selection of CNS samples and they are therefore not independent. In the top-50 comparisons, there were on average 2.05 genes that overlapped between any pair of tissues. The highest total of overlaps was observed between CNS and other tissues, and between the selection of all tissues ("ALL") against individual tissue types. In this study, biologically related tissues had a significantly higher overlap than the global mean overlap of tissue pairs (with population mean = 2.05 and SD = 1.98). For example, in the top 50 comparisons, spinal cord, encephalon, hippocampus, and central nervous system had overlaps significantly above population mean (p = 0.01) using a permutation test with 100,000 permutations. Also heart versus heart left ventricle and ovary versus testis had significantly higher overlap values (p = 0.05). In some of the cases, however, the overlap was significant but the biological relationship was unclear. For example, liver and lung both overlapped most with heart (p = 0.015 and p = 0.005, respectively), although they have very different biological functions. From this comparison alone, it was not possible to provide a general evidence to fully support this hypothesis. The main reason could be the heterogeneity of tissue types studied and the lack of an accepted measure to define biological relatedness between tissue types. In fact, only few subgroups of tissues were available which had obvious related biological functions. Despite the very large set of curated data used in this study (4604 AffymetrixMouse430 2.0 array hybridizations), it was not possible to compile data for more groups of biologically closely related tissues, such as different types of muscles, because too few independent data sets about these tissues were publicly available.

On average, the SD of expression within each tissue type increased 30% between probe sets of rank 1 and rank 20, and 43%, 54% and 67% between rank 1 and rank 50, 100 and 200, respectively (see Additional file 4). The above findings indicate that, for each tissue type, a specific set of approximately 10-20 candidate genes exists that has significantly smaller variance of expression across samples from this tissue. At the same time, in the suboptimal range of expression stability (ranks 20 to 50), for each tissue type several genes were found that also had stable expression in other tissue types. As shown in Additional file 4 however, these "suboptimal candidates" have SD of expression between 30% and 67% higher than the best candidates for each tissue type and therefore are expected to be of more limited utility as reference genes in these individual conditions.

To assess the feasibility of extrapolating candidate reference genes from related tissue types, we carried out a validation experiment on B-lymphocytes. For human B-lymphocytes, only 4 arrays were available in the human 47 k dataset at the time of experimentation. We therefore chose to work with an extended set of tissues that were the most closely related to B-lymphocytes as identified by clustering the Genevestigator anatomical profiles of 10 randomly chosen sets of 400 genes. 46 arrays covering three closely related tissue types (B-lymphocytes, 4 arrays; lymphoblast cells, 24 arrays; lymphocytes, 18 arrays) were selected. Six novel candidate reference genes proposed by Ref-Genes were selected for this study and were compared to five commonly used reference genes (SDHA, GAPDH, YWHAZ, B2 M, RPL13a). The RT-qPCR validation experiment was carried out on lymphoblastoid cell lines (LCLs) of 15 subjects. The results of the top 8 genes as selected by GeNorm are shown in Figure 3. Two of the candidate genes obtained from RefGenes performed best and yielded significantly lower M values in GeNorm than the other reference genes. The remaining RefGenes candidates were similarly or less stably expressed than the control reference genes. Although in the microarray data (comprising several tissue types) all candidates proposed by RefGenes were more stable than commonly used reference genes, in this particular experiment based on LCLs only, the ranking of variances was different. This illustrates that expanding the search to related tissues has the potential to yield significantly better candidates, but it may be necessary to test a larger number of candidates, as some of them may be of similar or lower quality than commonly used reference genes. It must be noted, however, that not only the variance, but also the expression intensity range should be considered in choosing a reference gene. In fact, the commonly used reference genes tested had lower Cq values (reflecting very high expression levels), and therefore the novel RefGenes candidates could be preferred if their Cq values are closer to those of a specific target gene and their variances are similar to alternative reference genes.

#### Hypothesis 4 (orthology clause)

Our fourth hypothesis was that the stability of expression of gene orthologs remains similar across related species. Here, we cannot provide a general proof of principle, but an initial set of evidence to substantiate this hypothesis.

As a case study, we checked whether orthologs of genes that are highly stable in mouse liver could be used as alternative reference genes for RT-qPCR experiments carried out on cattle liver and pig liver samples. In fact, although Genevestigator currently does not contain data from these species, we hypothesized that the positive results obtained with mouse liver could be reproduced in other species by choosing the corresponding orthologs. Due to the incompleteness of available annotations for orthologs across these species, from the four genes that were previously validated in mouse, two (GAK and VPS4A) were found in cattle and pig. We identified a further gene (PMPCA) that was stable in mouse microarray data and was available as an ortholog in cattle and pig. These three genes were compared to three commonly used reference genes (ACTB, GAPDH, and UBQ for cattle, and Histone H3, GAPDH and UBQ for pig) in a RT-qPCR experiment comprising 42 cattle liver samples and 48 pig liver samples. The application of both GeNorm and Normfinder to identify the most stable genes within the cattle dataset showed that the two best normalizers were GAK and VPS4A (Figure 3; see also Additional file 6). PMPCA performed similarly to commonly used reference genes. In pig, the extrapolation from mouse did not result in novel genes being significantly more stable than commonly used reference genes. In fact, expression stability was similar across most genes and was in a more narrow range as compared to the stability values obtained in other experiments (in the pig data, Avg M varied between 0.29 to 0.36). Histone H3, Ubiquitin and VPS4A performed best, followed by GAK, GAPDH and PMPCA. Concluding from the results of all three species, GAK and VPS4A seem to have a conserved expression stability and to be suitable candidates for normalizing RT-qPCR experiments on liver samples. Overall, our results show that genes that were highly stable in mouse liver had orthologs in other species that were also highly stable. In our experiments, they performed similarly or better than commonly used reference genes. This is particularly useful for those cases where the search for new reference genes is limited by the amount of microarray data available for a given species, but abundant data is available in related species.

### The RefGenes tool

**Figure 5:**
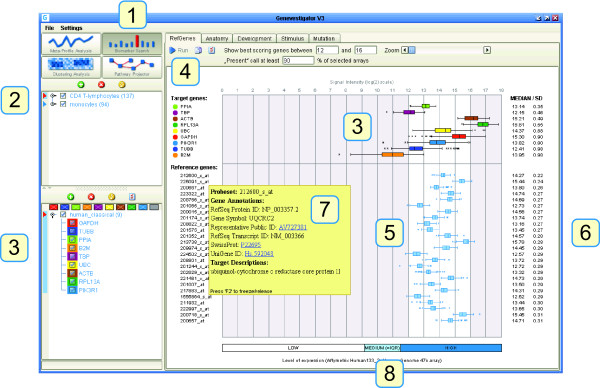
**Graphical user interface of the RefGenes tool in Genevestigator**. 1) The RefGenes tool belongs to the Biomarker Search toolset. 2) Panel for the selection of arrays associated with various experiments or conditions. 3) Panel for the selection of genes (target genes or commonly used reference genes for comparison). These genes are represented in the graph on the right with box and whiskers plots of signal intensity. In this example, the box and whiskers plots of expression in T-lymphocytes of 8 commonly used reference genes and a target gene (PIK3R1) are shown. 4) RefGenes toolbar, with fields to de fine the range of signal intensity within which new reference genes must be searched. The search for reference genes is triggered by a click on the "Run" button. 5) Box and whiskers plot of signal intensity of the new reference genes proposed by RefGenes. 6) The numerical values of the median and standard deviation of signal intensity are shown. 7) For each reference gene proposed by RefGenes, additional information is available in the mouse-over tooltip. 8) The typical range of low, medium, and high expression is shown for the array type chosen in (2). Medium is defined as the interquartile range (IQR).

Our results suggest that for RT-qPCR it is best to identify specific reference genes for each experiment individually. To this end, we have developed Ref-Genes, a novel online tool from the Genevestigator platform. The main feature of RefGenes is to search for genes that exhibit minimal expression variance across a chosen set of arrays. Its graphical user interface is shown in Figure 5. RefGenes is very simple to use and requires only two main actions:

1) choosing a set of microarrays (samples)

2) choosing the range of expression.

### Choosing a set of microarrays

The user can create selections of microarrays according to organism and to chosen sample properties, for example a set of human arrays from a particular tissue type. Currently, array selections can be done from sample annotations such as anatomical part, developmental stage, treatment, disease, genetic modification, or tumor type. Because the database is populated with a very large number of experiments, researchers can often identify subsets of arrays from a context similar to that from their own RT-qPCR experiment. Our recommendation is to select at least three independent studies comprising at least 60 arrays in total. If this cannot be reached within a specific context, it may be worth extending this context with closely related conditions. In the example described earlier with T-lymphocytes, we selected 137 arrays hybridized with transcripts from CD4 T-Lymphocyte samples.

### Choosing the range of expression

Theoretically, as long as data normalization is carried out in the linear range of amplification of both target and reference gene, it is not necessary for them to be in the same range of expression. However, some experimenters prefer using reference genes that are in a similar range of expression as their genes of interest. In RefGenes, the user can define the upper and lower bounds of the search space such as to obtain candidate reference genes within these bounds. As an additional information, a bar below the graph indicates, for a given microarray platform, the typical ranges of low, medium, and high expression (where "Medium" indicates the interquartile range). We recommend to upload genes of interest as well as alternative reference genes for a comparison with new candidates that will be proposed by RefGenes. In the screen shot shown in Figure 5, we uploaded the probe set identifiers for GAPDH, TUBB, PPIA, B2 M, TBP, UBC, ACTB, RPL13A, as well as that of PIK3R1 as an example of a target gene to be measured by RT-qPCR in CD4 T-lymphocytes. We then defined the range of reference gene expression to be slightly above and below that of PIK3R1.

### Searching for reference genes

The "Run" button allows to trigger the search algorithm based on the selections of arrays and genes. The Genevestigator engine searches for genes with the lowest variance within this selection of arrays and displays the top 25 probe sets. For each probe set, the mean and standard deviation are indicated. Mouse-over tooltips over each probe set provide additional information such as gene name and IDs for various gene models. In the present example, after launching the search by clicking on the "Run" button, RefGenes suggested 25 potential reference genes, of which the standard deviation of expression was between 0.22 and 0.31. As a comparison, the standard deviations of commonly used reference genes was between 0.35 and 0.98.

### Validating potential reference genes

The candidate reference genes obtained can be pre-validated by checking their expression across all microarrays available for that array type. The user can verify whether there are particular conditions in which their expression varies unexpectedly. For example, one can create a new selection of genes obtained in RefGenes, and go to the Meta-Profile Analysis toolset to check their expression levels in different tissues (*Anatomy *tool), or their response to different diseases, chemicals, hormones, etc. (*Conditions *tool). In general, genes proposed by Ref-Genes appear to be very unresponsive to a wide variety of conditions. In the example with CD4 T-lymphocytes, one of the genes was unlikely to be a good candidate as it responded strongly to a subset of conditions in the *Conditions *tool. We also observed that most of the candidate genes had a slight response to various tumors and to oncolytic viruses (see Additional file 8).

## Discussion

Our approach builds on previous studies showing that reference genes identified from microarray data often performed better in normalizing RT-qPCR experiments than commonly used reference genes. In contrast to previous studies, our approach combines three levels: 1) it searches for the most stable candidates from a genome-wide set of genes (rather than from a small set of commonly used reference genes), 2) it allows to restrict the search to an expression range similar to that of own target genes, and 3) it allows users to flexibly choose, from a very large array compendium, context-specific sets of microarrays based on sample annotations. Additionally, based on the Genevestigator standardized data content, it allows users to cross-validate new candidates across a large set of experimental conditions prior to testing them in the laboratory. RefGenes therefore allows to select experimental conditions that are similar to that of a specific experiment and to obtain reliable and condition-specific candidates for the normalization of RT-qPCR or other types of transcript quantification data. Although Genevestigator currently contains more than 50,000 arrays, several experimental conditions may not yet be well populated (e.g. B-lymphocytes). In such cases, it is recommended to include additional arrays from related experimental conditions or tissues.

In our approach, we are extrapolating results from a variety of microarray experiments carried out within a specific biological context (e.g. tissue type) to predict gene stability in similar contexts. We show across several RT-qPCR experiments that the extrapolation is generally reliable. Nevertheless, because we are comparing different sets of biological experiments as well as two technologies, results may differ between the two platforms. The main source of discrepancy is likely to be due to differences in the types of biological experiments and samples between the predictor dataset (microarray) and the target experiment (e.g. RT-qPCR). It is also possible that the candidates proposed by RefGenes are biased by the inherent nature of microarray data as compared to RT-qPCR data, or by data transformation procedures during normalization. In fact, one would expect variance to depend linearly on the mean based on original intensities (which are proportional to molecular concentration). Nevertheless, and despite differences in sensitivity between the two technologies, we did not observe major discrepancies that would question the use of microarray data to identify stably expressed genes to be used as references for RT-qPCR. In fact, the experiments described above, as well as previously published work, e.g., [18] demonstrate that the availability of quality controlled and normalized oligonucleotide microarray data (such as Affymetrix GeneChip arrays) allows to identify better reference gene candidates than commonly used reference genes. The use of different normalization methods or measures of variance is expected to influence the outcome of a search by RefGenes, but overall it is unlikely that genes that exhibit a high stability within a RT-qPCR experiment would not be identified by either of these methods at the microarray level. In particular, differences between popular algorithms, such as RMA and MAS5, are minor in the medium to high expression range for data from single experiments [30]. This is the range where most RT-qPCR normalization genes are located. When combining data from multiple experiments, the method used to correct for cross-experiment effects will have an additional influence on the overall variance. The same holds true for batch effects within a single experiment. Here, we show a proof of principle of reference gene identification using a data compendium normalized with MAS5 (cross-normalized with global scaling) and several RT-qPCR validation studies. A further measure to *in silico *validate candidates proposed by RefGenes is to check how they respond to different conditions using the *Conditions *and *Genotypes *tools in the Meta-Profile Analysis toolset. In general, stably expressed genes respond very weakly to internal or external perturbations (see for example Additional file 8 figure D). Batch and experimental biases are minor in this dataset since we are looking at (log)ratio values that were calculated from individual treatment versus control sets of samples from the same batch or experiment.

**Figure 6:**
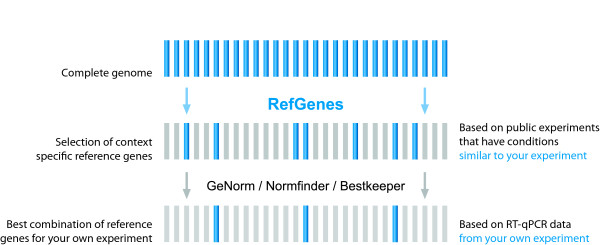
**Proposed approach for the selection of suitable reference genes**. Rather than starting from a subset of commonly used reference genes, we propose to start with an objective choice of candidate genes based on public microarray data obtained from similar experimental conditions. The second step remains identical, i.e. the validation of several candidates within the RT-qPCR experiment being carried out, and the selection of the most stable ones using algorithms such as GeNorm, Norm finder, or Bestkeeper.

In summary, for individual experimental conditions it is worth searching for a number of new candidates and validating them against commonly used reference genes. The proposed general approach is illustrated in Figure 6: instead of starting with a handful of commonly used reference genes, we propose to start with a statistically selected, context-specific set of candidate genes identified by Ref-Genes, and then to validate them (optionally together with commonly used genes) within the experiment under study using algorithms such as GeNorm, Norm finder, or Bestkeeper. We also strongly recommend researchers to read the MIQE guidelines [17] as a guide to help carrying out and publishing their work.

## Conclusions

We conclude that the identification of context-specific reference genes, combined with existing methods for normalization against multiple controls, is expected to significantly improve the quality and sensitivity of expression quantification experiments, facilitating the correct interpretation of RT-qPCR data. RefGenes is freely available for academic users (upon registration to prove one's affiliation), while for commercial users, RefGenes is available as part of a Genevestigator subscription. Ref Genes is a Genevestigator tool and is available at http://www.genevestigator.com.

## Methods

### Selection of reference genes

Data from Genevestigator was normalized, quality controlled, and annotated manually as described previously [29]. In brief, Affymetrix expression array data used for this study was normalized using the MAS5 algorithm, with global scaling set to a target value of 1000. The quality of the arrays was assessed using various Bioconductor [31] packages, including AffyQCReport and SimpleAffy [32]. Sample descriptions were annotated using the Genevestigator application ontologies for anatomical parts, stage of development, and experimental perturbations. Novel reference gene candidates used for experimental validation were obtained from RefGenes. The search algorithm identifies, for a chosen set of microarrays, those probe sets for which the standard deviation of signal intensities across these arrays is lowest.

In the below experiments, the set of commonly used reference genes was arbitrarily chosen from genes that had been previously used as references in the respective laboratories.

### RT-qPCR for mouse liver

16 liver samples were harvested from WT and Re-verb alpha mutant females fed with 2 different diets. RNA was extracted according to Fonjallaz's protocol [33]. cDNA was synthesized from 1 *μ*g of total RNA using random hexamers and Supercript II reverse transcriptase (Invitrogen) following suppliers instructions. SYBR green assays were designed using the program Primer Express v 2.0 (Applied Biosystems) with default parameters such that they spanned exon bondaries when possible. Amplicon sequences were aligned against the mouse genome by BLAST to check for specificity. Oligonucleotides were obtained from Invitrogen. The efficiency of each design was tested with serial dilutions of cDNA. PCR reactions (10 *μ*L volume) contained diluted cDNA, 2 × Power SYBR Green Master Mix (Applied Biosystems), 300 nM of forward and reverse primers. PCR were performed on a SDS 7900 HT instrument (Applied Biosystems) with the following parameters: 50°C for two minutes, 95°C for ten minutes, and 40 cycles at 95°C for 15 seconds and 60°C for one minute. Each reaction was performed in three replicates on a 384-wells plate. Raw Cq values obtained with SDS 2.2.2 software (Applied Biosystems) were analysed and the best house keeping genes selected according to the GeNorm method [13]. The forward (F) and reverse (R) primers used for this experiment were:

Mm GAK F CTGCCCACCAGGCATTTG

Mm GAK R CCATGTCACATACATATTCAATGTACCT

Mm MRPl46 F GGGAGCAGGCATTCCTACAG

Mm MRPl46 R GGTCCGGTCATTTTTTTTGTCA

Mm SRP72 F CACCCAGCAGACAGACAAACTG

Mm SRP72 R GCACTCATCGTAGCGTTCCA

Mm VPS4A F GACAACGTCAACCCTCCAGAAA

Mm VPS4A R TCTGTGGCTTTTGTCACCAGAT

Mm TUBB F GCAGTGCGGCAACCAGAT

Mm TUBB R AGTGGGATCAATGCCATGCT

Mm HPRT F GCTCGAGATGTCATGAAGGAGAT

Mm HPRT R AAAGAACTTATAGCCCCCCTTGA

Mm ACTB F CTAAGGCCAACCGTGAAAAGAT

Mm ACTB R CACAGCCTGGATGGCTACGT

Mm GAPDH F TCCATGACAACTTTGGCATTG

Mm GAPDH R CAGTCTTCTGGGTGGCAGTGA

### RT-qPCR for human LCLs

Human lymphocytes were isolated from blood samples by Ficoll Lymphocyte Separation Medium (MP Biochemicals). Lymphoblastoid cell lines were obtained by transformation of the fresh lymphocytes with Epstein-Barr Virus and grown in advanced RPMI medium supplemented with 2% fetal bovine serum, 2 mM glutaMAX (L-Alanyl-L-Glutamine), 50 units/mL penicillin, 50 *μ*g/mL streptomycin and 2% phytohemagglutinin, all from Invitrogen (Carlsbad, CA, USA). For extraction of total RNA, the transformed lymphoblastoid cell lines were harvested, lysed in RLT buffer (Qiagen, Valencia, CA, USA) and homogenized with a QIAshredder homogenizer (Qiagen). RNA purification was performed with the Qiagen RNeasy Plus Mini-kit (Qiagen) and RNA was quantified and checked for its purity using the Nanodrop spectrophotometer (Nanodrop Technologies, Wilmington, DE, USA). Reverse transcription was performed on 2 *μ*g total RNA with the superscript III first-strand synthesis system for RT-PCR kit (Invitrogen) using a mixture of oligo (dT)20 and random hexamer primers. Primer sequences for conventional reference genes were obtained from [13] and primer sequences for the novel candidate reference genes proposed by RefGenes were designed using primer 3 software [34] (see list below). 20 ng total RNA equivalents of cDNA were used in each RT-qPCR amplification run in triplicate. Detection of the PCR product was carried out by the LC480 real-time PCR detection system (Roche, Nutley, NJ, USA) using LightCycler 480 SYBR Green I Master mix and 250 nM primer. Relative quantities were calculated by the delta-Ct method and expression stability of the housekeeping genes was evaluated by GeNorm [13]. The primers used in this study were as follows:

Hs B2 M F TGCTGTCTCCATGTTTGATGTATCT

Hs B2 M R TCTCTGCTCCCCACCTCTAAGT

Hs GAPD F TGCACCACCAACTGCTTAGC

Hs GAPD R GGCATGGACTGTGGTCATGAG

Hs RPL13A F CCTGGAGGAGAAGAGGAAAGAGA

Hs RPL13A R TTGAGGACCTCTGTGTATTTGTCAA

Hs SDHA F TGGGAACAAGAGGGCATCTG

Hs SDHA R CCACCACTGCATCAAATTCATG

Hs YWHAZ F ACTTTTGGTACATTGTGGCTTCAA

Hs YWHAZ R CCGCCAGGACAAACCAGTAT

Hs BUD13 F GATGGAGATTTGCCTGTGGT

Hs BUD13 R ATTTGGCACTGGAACGAAAG

Hs EIF4EBP2 F TAGCCCTGGCACCTTAATTG

Hs EIF4EBP2 R AACTGAGCATCATCCCCAAC

Hs GOLT1B F CCTTATTGGTTGGCCTTTGA

Hs GOLT1B R AGCCAACAACGACAGGAAAG

Hs INTS4 F GCAGCTCCATGAAAGAGGAC

Hs INTS4 R ACCCAGATAAGCTGGACTGC

Hs SAP130 F GAGGCCAGTTTCTGCAGTTC

Hs SAP130 R GCACCAGGTGGTAGGTCACT

Hs TATDN2 F ACAAATGCTCTCCACCCCTA

Hs TATDN2 R TCCATCACCACCTCCCTATC

Hs ZNF410 F CTCCGAAAACATCTGGTGGT

Hs ZNF410 R CTGCAGGTGATGCTTTCTCA

### RT-qPCR for cattle and pig liver

Immediately after slaughtering pieces of liver tissue were taken from calves and piglets fed different dietary fiber diets and snap frozen in liquid nitrogen. Total RNA was extracted with TriFast reagent (Peqlab, Erlangen, Germany) according to the manufacturer's instructions. RNA quantity and quality were assessed using a NanoDrop spectrophotometer (Peqlab, Erlangen, Germany) and a Bioanalyzer 2100 with RNA Nano Chips (Agilent Technologies, Palo Alto, CA). RNA integrity ranged between 7.2 and 8.4 and OD260/280 between 1.81 and 1.96. Samples were diluted to a working concentration of 10 ng/*μ*L. Primers were chosen for cattle and pig orthologs of mouse genes identified as stably expressed in liver tissue. Primer design was done using the primer 3 software [26] and primers were purchased from Eurofins MWG Operon (Ebersberg, Germany). One-step RT-qPCR (gene specific reverse transcription immediately followed by RT-qPCR) was performed using SuperScirpt III Platinum SYBR Green One-Step qRT-PCR kit (Invitrogen, Carlsbad, CA). PCR temperature profiles were optimized for each primer pair and identity of amplicons was verified by sequencing (Sequencing Service, Ludwig Maximilians Universitaet, Munich). Signal detection was achieved with a Rotor-Gene 3000 (Corbett Life Sciences, Sydney, Australia). Validation of the housekeeping genes was done by GenEx Professional Software ver. 4.4.2 (multiD Analyses AB, Gothenburg, Sweden) utilizing GeNorm and Normfinder. Below are the primers used for this study:

Bovine primers:

Bt ACTB F AACTCCATCATGAAGTGTGACG

Bt ACTB R GATCCACATCTGCTGGAAGG

Bt GAPDH F GTCTTCACTACCATGGAGAAGG

Bt GAPDH R TCATGGATGACCTTGGCCAG

Bt UBQ F AGATCCAGGATAAGGAAGGCAT

Bt UBQ R GCTCCACCTCCAGGGTGAT

Bt VPS4A F CAAAGCCAAGGAGAGCATTC

Bt VPS4A R ATGTTGGGCTTCTCCATCAC

Bt GAK F TCTGGGAAGTGGCAGAGAGT

Bt GAK R CGGCACGTCTGGTAGAAGAT

Bt PMPCA F CATCCCAGAATAAGTTTGGACAG

Bt PMPCA R AGAATCAGCAGACACAGCATACA

Porcine primers:

Ss UBIQ F AGATCCAGGATAAGGAAGGCAT

Ss UBIQ R GCTCCACCTCCAGGGTGAT

Ss Histon H3 F ACTGGCTACAAAAGCCGCTC

Ss Histon H3 R ACTTGCCTCCTGCAAAGCAC

Ss GAPDH F AGCAATGCCTCCTGTACCAC

Ss GAPDH R AAGCAGGGATGATGTTCTGG

Ss GAK F AATCGCAGTGATGTCCTTCC

Ss GAK R GCTTCGAGTCCAGAAACAGC

Ss VPS4A F CAAAGCCAAGGAGAGCATTC

Ss VPS4A R ATGTTGGGCTTCTCCATCAC

Ss PMPCA F CATCCCAGAATAAGTTTGGACAG

Ss PMPCA r AGAATCAGCAGACACAGCATACA

### RT-qPCR for Arabidopsis tissues

Total RNA was isolated from 5 day old seedlings or from 15 day old leaves following the TRIzol protocol (Invitrogen). RNA quantity and quality was assayed via spectrophotometer analysis (Pharmacia Biotech). First-strand cDNA synthesis was performed with 3 *μ*g of total RNA using SuperScript II RNase H-reverse transcriptase (Invitrogen) and oligo-dT primers (Fermentas) according to the manufacturer's instructions. The 20-*μ*L cDNA reaction was diluted 1:100 with deionized water, and 4 *μ*L were used for each RT-PCR amplification. Amplifications were performed as technical duplicates and biological quadruplicates in 96-well plates in a 20-*μ*L reaction volume containing 10 *μ*L 2× Fast SYBR Green qPCR MasterMix (Applied Biosystem). Reactions were performed on a 7500 Fast Real-Time PCR System (Applied Biosystems). Primers for all amplifications, designed with PerlPrimer v1.1.10 (freeware by Owen Marshall), were located on exon-exon borders to prevent amplification of potentially contaminating genomic DNA.

Primers used for Arabidopsis seedlings:

At At3g24160 F ATATCAGACAGGCAGTCAGCG

AT At3g24160 R TGCTAAAGCATCGATACCACC

At At3g27820 F GCGGTGGCTATATCGGTATGG

At At3g27820 R AAAGAGACGTGCCATGCAGTG

At At1g13320 F CAAGTGAACCAGGTTATTGGGA

At At1g13320 R ATAGCCAGACGTACTCTCCAG

Primers used for Arabidopsis leaves:

At At3g61710 F AGACACAGGTTGAACAGCCA

At At3g61710 R GTATGCTTCCACGTCCCTCG

At At1g32050 F TCACCTACTTGATTCACATTGGCT

At At1g32050 R ATCAATTGCTGCAAGCACAC

At At3g01150 F CCACCGGAGCAGAGATTACAC

At At3g01150 R CAACTTTCTTGCCGTCAGCAC

Primers used for Arabidopsis shoot apices:

At At3G17920 F AACGACACTGTCAGATTCCA

At At3g17920 R CTACTTCCCGTTGCTTATAGGTG

At At2G17390 F CAGACTGTTGCAGCTGAACCT

At At2g17390 R GCTTTCAAACCCTCGACATCAC

At At5G51880 F CAGTATTGTAGCTGAGGTAGCTCC

At At5g51880 R CGCCTTTGGAGACATTCCTC

## Authors' contributions

TH, MW, SB, OL, WG and PZ elaborated the concepts, designed the tool, developed the software, and curated data. Validation experiments using RT-qPCR were performed with mouse liver samples by MD and PD, on cattle and pig liver by SM and MP, on Arabidopsis tissues by LB, and on human B-lymphocytes by PV and LK. All authors were involved in writing the manuscript. All authors read and approved the final draft.

## Supplementary Material

Additional file 1 **List of publications related to reference gene validation**. Publications that report about the validation of small sets of commonly used reference genes for various biological contexts. Click here for file

Additional file 2 **Variance of gene expression across different array types**. Standard deviation of signal intensity versus mean signal intensity for all probe sets from different Affymetrix array types available in Genevestigator. Click here for file

Additional file 3 **Variance of gene expression across different tissue types**. Standard deviation of the top 50 probe sets that were most stable across all conditions (5014 samples from the AffymetrixHuman133 2.0 platform) across 186 different tissue categories. Click here for file

Additional file 4 **Ranking of the SD of the most stable probe sets across different mouse tissues**. Ranking of the SD of the most stable probe sets identified for a variety of mouse tissue samples (AffymetrixMouse430 2.0 platform). Click here for file

Additional file 5 **Ranks of the SD of commonly used reference genes across different mouse tissues**. Ranks of the SD across different tissues of probe sets representing commonly used reference genes in mouse (AffymetrixMouse430 2.0 platform). Click here for file

Additional file 6 **GeNorm calculations**. This figure shows the complete set of GeNorm calculations for the results summarized in Figure 3 of the article. Click here for file

Additional file 7 **Original measurement data of RT-qPCR validation experiments**. For each validation experiment, the original Cq values for each sample are provided. Click here for file

Additional file 8 **Pre-validation of reference genes for CD4 T-lymphocytes**. This figure shows screen shots of meta-profile data for candidate reference genes for CD4 T-lymphocytes. Click here for file
