# Supplementary material for: RefGenes: identification of reliable and condition specific reference genes for RT-qPCR data normalization
Source: BMC Genomics. 2011 Mar 21;12:156. doi: 10.1186/1471-2164-12-156 (PMC3072958; doi:10.1186/1471-2164-12-156)
Supplement: Additional file 1 — List of publications related to reference gene validation. Publications that report about the validation of small sets of commonly used reference genes for various biological contexts. [file 1471-2164-12-156-S1.PDF]

Additional file 1

Publications reporting the validation of small sets of commonly used reference genes for various contexts. [1–97]

References

1. Ahn K, Huh JW, Park SJ, Kim DS, Ha HS, Kim YJ, Lee JR, Chang KT, Kim HS: **Selection of internal reference genes for SYBR green qRT-PCR studies of rhesus monkey (Macaca mulatta) tissues**. *BMC Mol. Biol.* 2008, **9**:78.
2. Al-Bader MD, Al-Sarraf HA: **Housekeeping gene expression during fetal brain development in the rat-validation by semi-quantitative RT-PCR**. *Brain Res. Dev. Brain Res.* 2005, **156**:38–45.
3. Allen D, Winters E, Kenna PF, Humphries P, Farrar GJ: **Reference gene selection for real-time rtPCR in human epidermal keratinocytes**. *J. Dermatol. Sci.* 2008, **49**:217–225.
4. Araya MT, Siah A, Mateo D, Markham F, McKenna P, Johnson G, Berthe FC: **Selection and evaluation of housekeeping genes for haemocytes of soft-shell clams (Mya arenaria) challenged with Vibrio splendidus**. *J. Invertebr. Pathol.* 2008, **99**:326–331.
5. Aswal AP, Raghav S, De S, Thakur M, Goswami SL, Datta TK: **Expression stability of two housekeeping genes (18S rRNA and G3PDH) during in vitro maturation of follicular oocytes in buffalo (Bubalus bubalis)**. *Anim. Reprod. Sci.* 2008, **103**:164–171.
6. Balogh A, Paragh G, Juhasz A, Koebling T, Toerocsik D, Miko E, Varga V, Emri G, Horkay I, Scholtz B, Remenyik E: **Reference genes for quantitative real time PCR in UVB irradiated keratinocytes**. *J. Photochem. Photobiol. B, Biol.* 2008, **93**:133–139.
7. Barber RD, Harmer DW, Coleman RA, Clark BJ: **GAPDH as a housekeeping gene: analysis of GAPDH mRNA expression in a panel of 72 human tissues**. *Physiol. Genomics* 2005, **21**:389–395.
8. Barsalobres-Cavallari CF, Severino FE, Maluf MP, Maia IG: **Identification of suitable internal control genes for expression studies in Coffea arabica under different experimental conditions**. *BMC Mol. Biol.* 2009, **10**:1.
9. Biederman J, Yee J, Cortes P: **Validation of internal control genes for gene expression analysis in diabetic glomerulosclerosis**. *Kidney Int.* 2004, **66**:2308–2314.
10. Boda E, Pini A, Hoxha E, Parolisi R, Tempia F: **Selection of reference genes for quantitative real-time RT-PCR studies in mouse brain**. *J. Mol. Neurosci.* 2009, **37**:238–253.
11. Bohle K, Jungebloud A, Goecke Y, Dalpiaz A, Cordes C, Horn H, Hempel DC: **Selection of reference genes for normalisation of specific gene quantification data of Aspergillus niger**. *J. Biotechnol.* 2007, **132**:353–358.
12. Bonefeld BE, Elfving B, Wegener G: **Reference genes for normalization: a study of rat brain tissue**. *Synapse* 2008, **62**:302–309.
13. Bonnet-Duquennoy M, Abaibou H, Tailhardat M, Lazou K, Bosset S, Le Varlet B, Cleuziat P, Kurfuerst R: **Study of housekeeping gene expression in human keratinocytes using OLISA, a long-oligonucleotide microarray and q RT-PCR**. *Eur J Dermatol* 2006, **16**:136–140.
14. Calcagno AM, Chewning KJ, Wu CP, Ambudkar SV: **Plasma membrane calcium ATPase (PMCA4): a housekeeper for RT-PCR relative quantification of polytopic membrane proteins**. *BMC Mol. Biol.* 2006, **7**:29.
15. Campos MS, Rodini CO, Pinto-Junior DS, Nunes FD: **GAPD and tubulin are suitable internal controls for qPCR analysis of oral squamous cell carcinoma cell lines**. *Oral Oncol.* 2009, **45**:121–126.
16. Cappelli K, Felicetti M, Capomaccio S, Spinsanti G, Silvestrelli M, Supplizi AV: **Exercise induced stress in horses: selection of the most stable reference genes for quantitative RT-PCR normalization**. *BMC Mol. Biol.* 2008, **9**:49.
17. Carrillo-Casas EM, Hernandez-Castro R, Suarez-Gueemes F, de la Pena-Moctezuma A: **Selection of the internal control gene for real-time quantitative rt-PCR assays in temperature treated Leptospira**. *Curr. Microbiol.* 2008, **56**:539–546.
18. Cicinnati VR, Shen Q, Sotiropoulos GC, Radtke A, Gerken G, Beckebaum S: **Validation of putative reference genes for gene expression studies in human hepatocellular carcinoma using real-time quantitative RT-PCR**. *BMC Cancer* 2008, **8**:350.
19. Cook NL, Vink R, Donkin JJ, van den Heuvel C: **Validation of reference genes for normalization of real-time quantitative RT-PCR data in traumatic brain injury**. *J. Neurosci. Res.* 2009, **87**:34–41.

20. Coulson DT, Brockbank S, Quinn JG, Murphy S, Ravid R, Irvine GB, Johnston JA: **Identification of valid reference genes for the normalization of RT qPCR gene expression data in human brain tissue**. *BMC Mol. Biol.* 2008, **9**:46.

21. Cui X, Zhou J, Qiu J, Johnson MR, Mrug M: **Validation of Endogenous Internal Real-Time PCR Controls in Renal Tissues**. *Am. J. Nephrol.* 2009, **30**:413–417.

22. de Boer ME, de Boer TE, Marien J, Timmermans MJ, Nota B, van Straalen NM, Ellers J, Roelofs D: **Reference genes for QRT-PCR tested under various stress conditions in Folsomia candida and Orchesella cincta (Insecta, Collembola)**. *BMC Mol. Biol.* 2009, **10**:54.

23. De Boever S, Vangestel C, De Backer P, Croubels S, Sys SU: **Identification and validation of housekeeping genes as internal control for gene expression in an intravenous LPS inflammation model in chickens**. *Vet. Immunol. Immunopathol.* 2008, **122**:312–317.

24. de Kok JB, Roelofs RW, Giesendorf BA, Pennings JL, Waas ET, Feuth T, Swinkels DW, Span PN: **Normalization of gene expression measurements in tumor tissues: comparison of 13 endogenous control genes**. *Lab. Invest.* 2005, **85**:154–159.

25. Derks NM, Mueller M, Gaszner B, Tilburg-Ouwens DT, Roubos EW, Kozicz LT: **Housekeeping genes revisited: different expressions depending on gender, brain area and stressor**. *Neuroscience* 2008, **156**:305–309.

26. Dhar AK, Bowers RM, Licon KS, Veazey G, Read B: **Validation of reference genes for quantitative measurement of immune gene expression in shrimp**. *Mol. Immunol.* 2009, **46**:1688–1695.

27. Doroudi R, Andersson M, Svensson PA, Ekman M, Jern S, Karlsson L: **Methodological studies of multiple reference genes as endogenous controls in vascular gene expression studies**. *Endothelium* 2005, **12**:215–223.

28. Drury S, Anderson H, Dowsett M: **Selection of REFERENCE genes for normalization of qRT-PCR data derived from FFPE breast tumors**. *Diagn. Mol. Pathol.* 2009, **18**:103–107.

29. Exposito-Rodriguez M, Borges AA, Borges-Perez A, Perez JA: **Selection of internal control genes for quantitative real-time RT-PCR studies during tomato development process**. *BMC Plant Biol.* 2008, **8**:131.

30. Falco G, Stanghellini I, Ko MS: **Use of Chuk as an internal standard suitable for quantitative RT-PCR in mouse preimplantation embryos**. *Reprod. Biomed. Online* 2006, **13**:394–403.

31. Fink T, Lund P, Pilgaard L, Rasmussen JG, Duroux M, Zachar V: **Instability of standard PCR reference genes in adipose-derived stem cells during propagation, differentiation and hypoxic exposure**. *BMC Mol. Biol.* 2008, **9**:98.

32. Fernandes JM, Mommens M, Hagen O, Babiak I, Solberg C: **Selection of suitable reference genes for real-time PCR studies of Atlantic halibut development**. *Comp. Biochem. Physiol. B, Biochem. Mol. Biol.* 2008, **150**:23–32.

33. Garcia-Crespo D, Juste RA, Hurtado A: **Selection of ovine housekeeping genes for normalisation by real-time RT-PCR; analysis of PrP gene expression and genetic susceptibility to scrapie**. *BMC Vet. Res.* 2005, **1**:3.

34. Goncalves S, Cairney J, Maroco J, Oliveira MM, Miguel C: **Evaluation of control transcripts in real-time RT-PCR expression analysis during maritime pine embryogenesis**. *Planta* 2005, **222**:556–563.

35. Gonzalez-Verdejo CI, Die JV, Nadal S, Jimenez-Marin A, Moreno MT, Roman B: **Selection of housekeeping genes for normalization by real-time RT-PCR: analysis of Or-MYB1 gene expression in Orobanche ramosa development**. *Anal. Biochem.* 2008, **379**:176–181.

36. Hibbeler S, Scharsack JP, Becker S: **Housekeeping genes for quantitative expression studies in the three-spined stickleback Gasterosteus aculeatus**. *BMC Mol. Biol.* 2008, **9**:18.

37. Ho-Pun-Cheung A, Bascoul-Mollevi C, Assenat E, Bibeau F, Boissiere-Michot F, Cellier D, Ychou M, Lopez-Crapez E: **Validation of an appropriate reference gene for normalization of reverse transcription-quantitative polymerase chain reaction data from rectal cancer biopsies**. *Anal. Biochem.* 2009, **388**:348–350.

38. Hoogewijs D, Houthoofd K, Matthijssens F, Vandesompele J, Vanfleteren JR: **Selection and validation of a set of reliable reference genes for quantitative sod gene expression analysis in C. elegans**. *BMC Mol. Biol.* 2008, **9**:9.

39. Infante C, Matsuoka MP, Asensio E, Canavate JP, Reith M, Manchado M: **Selection of housekeeping genes for gene expression studies in larvae from flatfish using real-time PCR**. *BMC Mol. Biol.* 2008, **9**:28.

40. Ingerslev HC, Pettersen EF, Jakobsen RA, Petersen CB, Wergeland HI: **Expression profiling and validation of reference gene candidates in immune relevant tissues and cells from Atlantic salmon (Salmo salar L.)**. *Mol. Immunol.* 2006, **43**:1194–1201.

41. Ishii T, Wallace AM, Zhang X, Gosselink J, Abboud RT, English JC, Pare PD, Sandford AJ: **Stability of housekeeping genes in alveolar macrophages from COPD patients**. *Eur. Respir. J.* 2006, **27**:300–306.

42. Jiang HB, Liu YH, Tang PA, Zhou AW, Wang JJ: **Validation of endogenous reference genes for insecticide-induced and developmental expression profiling of Liposcelis bostsrychophila (Psocoptera: Liposcelididae)**. *Mol. Biol. Rep.* 2009.

43. Janovick-Guretzky NA, Dann HM, Carlson DB, Murphy MR, Loor JJ, Drackley JK: **Housekeeping gene expression in bovine liver is affected by physiological state, feed intake, and dietary treatment**. *J. Dairy Sci.* 2007, **90**:2246–2252.

44. Jung M, Ramankulov A, Roigas J, Johannsen M, Ringsdorf M, Kristiansen G, Jung K: **In search of suitable reference genes for gene expression studies of human renal cell carcinoma by real-time PCR**. *BMC Mol. Biol.* 2007, **8**:47.

45. Kriegova E, Arakelyan A, Fillerova R, Zatloukal J, Mrazek F, Navratilova Z, Kolek V, du Bois RM, Petrek M: **PSMB2 and RPL32 are suitable denominators to normalize gene expression profiles in bronchoalveolar cells**. *BMC Mol. Biol.* 2008, **9**:69.

46. Langnaese K, John R, Schweizer H, Ebmeyer U, Keilhoff G: **Selection of reference genes for quantitative real-time PCR in a rat asphyxial cardiac arrest model**. *BMC Mol. Biol.* 2008, **9**:53.

47. Lee KS, Alvarenga TA, Guindalini C, Andersen ML, Castro RM, Tufik S: **Validation of commonly used reference genes for sleep-related gene expression studies**. *BMC Mol. Biol.* 2009, **10**:45.

48. Li YL, Ye F, Hu Y, Lu WG, Xie X: **Identification of suitable reference genes for gene expression studies of human serous ovarian cancer by real-time polymerase chain reaction**. *Anal. Biochem.* 2009, **394**:110–116.

49. Lisowski P, Pierzchała M, Gościk J, Pareek CS, Zwierzchowski L: **Evaluation of reference genes for studies of gene expression in the bovine liver, kidney, pituitary, and thyroid**. *J. Appl. Genet.* 2008, **49**:367–372.

50. Lyng MB, Laenkholm AV, Pallisgaard N, Ditzel HJ: **Identification of genes for normalization of real-time RT-PCR data in breast carcinomas**. *BMC Cancer* 2008, **8**:20.

51. Maron JL, Arya MA, Seefeld KJ, Peter I, Bianchi DW, Johnson KL: **pH but not hypoxia affects neonatal gene expression: relevance for housekeeping gene selection**. *J. Matern. Fetal. Neonatal. Med.* 2008, **21**:443–447.

52. McCurley AT, Callard GV: **Characterization of housekeeping genes in zebrafish: male-female differences and effects of tissue type, developmental stage and chemical treatment**. *BMC Mol. Biol.* 2008, **9**:102.

53. Mori R, Wang Q, Danenberg KD, Pinski JK, Danenberg PV: **Both beta-actin and GAPDH are useful reference genes for normalization of quantitative RT-PCR in human FFPE tissue samples of prostate cancer**. *Prostate* 2008, **68**:1555–1560.

54. Meller M, Vadachkoria S, Luthy D, Williams M: **Evaluation of housekeeping genes in placental comparative expression studies**. *Placenta* 2005, **26**:601–607.

55. Neuvians TP, Gashaw I, Sauer CG, von Ostau C, Kliesch S, Bergmann M, Haecker A, Grobholz R: **Standardization strategy for quantitative PCR in human seminoma and normal testis**. *J. Biotechnol.* 2005, **117**:163–171.

56. Nguewa PA, Agorreta J, Blanco D, Lozano MD, Gomez-Roman J, Sanchez BA, Valles I, Pajares MJ, Pio R, Rodriguez MJ, Montuenga LM, Calvo A: **Identification of importin 8 (IPO8) as the most accurate reference gene for the clinicopathological analysis of lung specimens**. *BMC Mol. Biol.* 2008, **9**:103.

57. Nicot N, Hausman JF, Hoffmann L, Evers D: **Housekeeping gene selection for real-time RT-PCR normalization in potato during biotic and abiotic stress**. *J. Exp. Bot.* 2005, **56**:2907–2914.

58. Nielsen KK, Boye M: **Real-time quantitative reverse transcription-PCR analysis of expression stability of Actinobacillus pleuropneumoniae housekeeping genes during in vitro growth under iron-depleted conditions**. *Appl. Environ. Microbiol.* 2005, **71**:2949–2954.

59. Ohl F, Jung M, Radoniae A, Sachs M, Loening SA, Jung K: **Identification and validation of suitable endogenous reference genes for gene expression studies of human bladder cancer**. *J. Urol.* 2006, **175**:1915–1920.

60. Ohl F, Jung M, Xu C, Stephan C, Rabien A, Burkhardt M, Nitsche A, Kristiansen G, Loening SA, Radoniae A, Jung K: **Gene expression studies in prostate cancer tissue: which reference gene should be selected for normalization?** *J. Mol. Med.* 2005, **83**:1014–1024.

61. Olbrich M, Gerstner E, Welzl G, Fleischmann F, Osswald W, Bahnweg G, Ernst D: **Quantification of mRNAs and housekeeping gene selection for quantitative real-time RT-PCR normalization in European beech (Fagus sylvatica L.) during abiotic and biotic stress**. *Z. Naturforsch., C, J. Biosci.* 2008, **63**:574–582.

62. Olsvik PA, Softeland L, Lie KK: **Selection of reference genes for qRT-PCR examination of wild populations of Atlantic cod Gadus morhua**. *BMC Res Notes* 2008, **1**:47.

63. Passmore M, Nataatmadja M, Fraser JF: **Selection of reference genes for normalisation of real-time RT-PCR in brain-stem death injury in Ovis aries**. *BMC Mol. Biol.* 2009, **10**:72.

64. Perez R, Tupac-Yupanqui I, Dunner S: **Evaluation of suitable reference genes for gene expression studies in bovine muscular tissue**. *BMC Mol. Biol.* 2008, **9**:79.

65. Perez S, Royo LJ, Astudillo A, Escudero D, Alvarez F, Rodriguez A, Gomez E, Otero J: **Identifying the most suitable endogenous control for determining gene expression in hearts from organ donors**. *BMC Mol. Biol.* 2007, **8**:114.

66. Piana C, Wirth M, Gerbes S, Viernstein H, Gabor F, Toegel S: **Validation of reference genes for qPCR studies on Caco-2 cell differentiation**. *Eur J Pharm Biopharm* 2008, **69**:1187–1192.

67. Pohjanvirta R, Niittynen M, Linden J, Boutros PC, Moffat ID, Okey AB: **Evaluation of various housekeeping genes for their applicability for normalization of mRNA expression in dioxin-treated rats**. *Chem. Biol. Interact.* 2006, **160**:134–149.

68. Remans T, Smeets K, Opdenakker K, Mathijsen D, Vangronsveld J, Cuypers A: **Normalisation of real-time RT-PCR gene expression measurements in Arabidopsis thaliana exposed to increased metal concentrations**. *Planta* 2008, **227**:1343–1349.

69. Rhinn H, Marchand-Leroux C, Croci N, Plotkine M, Scherman D, Escriou V: **Housekeeping while brain's storming Validation of normalizing factors for gene expression studies in a murine model of traumatic brain injury**. *BMC Mol. Biol.* 2008, **9**:62.

70. Ritz M, Garenaux A, Berge M, Federighi M: **Determination of rpoA as the most suitable internal control to study stress response in C. jejuni by RT-qPCR and application to oxidative stress**. *J. Microbiol. Methods* 2009, **76**:196–200.

71. Rodriguez-Mulero S, Montanya E: **Selection of a suitable internal control gene for expression studies in pancreatic islet grafts**. *Transplantation* 2005, **80**:650–652.

72. Romanowski T, Sikorska K, Bielawski KP: **GUS and PMM1 as suitable reference genes for gene expression analysis in the liver tissue of patients with chronic hepatitis**. *Med. Sci. Monit.* 2008, **14**:R147–152.

73. Rubie C, Kempf K, Hans J, Su T, Tilton B, Georg T, Brittner B, Ludwig B, Schilling M: **Housekeeping gene variability in normal and cancerous colorectal, pancreatic, esophageal, gastric and hepatic tissues**. *Mol. Cell. Probes* 2005, **19**:101–109.

74. Said HM, Hagemann C, Stojic J, Schoemig B, Vince GH, Flentje M, Roosen K, Vordermark D: **GAPDH is not regulated in human glioblastoma under hypoxic conditions**. *BMC Mol. Biol.* 2007, **8**:55.

75. Santos AR, Duarte CB: **Validation of internal control genes for expression studies: effects of the neurotrophin BDNF on hippocampal neurons**. *J. Neurosci. Res.* 2008, **86**:3684–3692.

76. Schlotter YM, Veenhof EZ, Brinkhof B, Rutten VP, Spee B, Willemse T, Penning LC: **A GeNorm algorithm-based selection of reference genes for quantitative real-time PCR in skin biopsies of healthy dogs and dogs with atopic dermatitis**. *Vet. Immunol. Immunopathol.* 2009, **129**:115–118.

77. Siah A, Dohoo C, McKenna P, Delaporte M, Berthe FC: **Selecting a set of housekeeping genes for quantitative real-time PCR in normal and tetraploid haemocytes of soft-shell clams, Mya arenaria**. *Fish Shellfish Immunol.* 2008, **25**:202–207.

78. Silveira ED, Alves-Ferreira M, Guimaraes LA, da Silva FR, Carneiro VT: **Selection of reference genes for quantitative real-time PCR expression studies in the apomictic and sexual grass Brachiaria brizantha**. *BMC Plant Biol.* 2009, **9**:84.

79. Silver N, Best S, Jiang J, Thein SL: **Selection of housekeeping genes for gene expression studies in human reticulocytes using real-time PCR**. *BMC Mol. Biol.* 2006, **7**:33.

80. Sirakov M, Zarrella I, Borra M, Rizzo F, Biffali E, Arnone MI, Fiorito G: **Selection and validation of a set of reliable reference genes for quantitative RT-PCR studies in the brain of the Cephalopod Mollusc Octopus vulgaris**. *BMC Mol. Biol.* 2009, **10**:70.

81. Spinsanti G, Panti C, Bucalossi D, Marsili L, Casini S, Frati F, Fossi MC: **Selection of reliable reference genes for qRT-PCR studies on cetacean fibroblast cultures exposed to OCs, PBDEs, and 17beta-estradiol**. *Aquat. Toxicol.* 2008, **87**:178–186.

82. Spinsanti G, Panti C, Lazzeri E, Marsili L, Casini S, Frati F, Fossi CM: **Selection of reference genes for quantitative RT-PCR studies in striped dolphin (Stenella coeruleoalba) skin biopsies**. *BMC Mol. Biol.* 2006, **7**:32.

83. Steinau M, Rajeevan MS, Unger ER: **DNA and RNA references for qRT-PCR assays in exfoliated cervical cells**. *J Mol Diagn* 2006, **8**:113–118.

84. Svobodova K, Bilek K, Knoll A: **Verification of reference genes for relative quantification of gene expression by real-time reverse transcription PCR in the pig**. *J. Appl. Genet.* 2008, **49**:263–265.

85. Tasara T, Stephan R: **Evaluation of housekeeping genes in Listeria monocytogenes as potential internal control references for normalizing mRNA expression levels in stress adaptation models using real-time PCR**. *FEMS Microbiol. Lett.* 2007, **269**:265–272.

86. Tatsumi K, Ohashi K, Taminishi S, Okano T, Yoshioka A, Shima M: **Reference gene selection for real-time RT-PCR in regenerating mouse livers**. *Biochem. Biophys. Res. Commun.* 2008, **374**:106–110.

87. Tang R, Dodd A, Lai D, McNabb WC, Love DR: **Validation of zebrafish (Danio rerio) reference genes for quantitative real-time RT-PCR normalization**. *Acta Biochim. Biophys. Sin. (Shanghai)* 2007, **39**:384–390.

88. Tong Z, Gao Z, Wang F, Zhou J, Zhang Z: **Selection of reliable reference genes for gene expression studies in peach using real-time PCR**. *BMC Mol. Biol.* 2009, **10**:71.

89. Valente V, Teixeira SA, Neder L, Okamoto OK, Oba-Shinjo SM, Marie SK, Scrideli CA, Paco-Larson ML, Carlotti CG: **Selection of suitable housekeeping genes for expression analysis in glioblastoma using quantitative RT-PCR**. *BMC Mol. Biol.* 2009, **10**:17.

90. Van Hiel MB, Van Wielendaele P, Temmerman L, Van Soest S, Vuerinckx K, Huybrechts R, Broeck JV, Simonet G: **Identification and validation of housekeeping genes in brains of the desert locust Schistocerca gregaria under different developmental conditions**. *BMC Mol. Biol.* 2009, **10**:56.

91. van Wijngaarden P, Brereton HM, Coster DJ, Williams KA: **Stability of housekeeping gene expression in the rat retina during exposure to cyclic hyperoxia**. *Mol. Vis.* 2007, **13**:1508–1515.

92. Verma AS, Shapiro BH: **Sex-dependent expression of seven housekeeping genes in rat liver**. *J. Gastroenterol. Hepatol.* 2006, **21**:1004–1008.

93. Wood SH, Clements DN, McEwan NA, Nuttall T, Carter SD: **Reference genes for canine skin when using quantitative real-time PCR**. *Vet. Immunol. Immunopathol.* 2008, **126**:392–395.

94. Xing W, Deng M, Zhang J, Huang H, Dirsch O, Dahmen U: **Quantitative evaluation and selection of reference genes in a rat model of extended liver resection**. *J Biomol Tech* 2009, **20**:109–115.

95. Yan HZ, Liou RF: **Selection of internal control genes for real-time quantitative RT-PCR assays in the oomycete plant pathogen Phytophthora parasitica**. *Fungal Genet. Biol.* 2006, **43**:430–438.

96. Zhang YW, Davis EG, Bai J: **Determination of internal control for gene expression studies in equine tissues and cell culture using quantitative RT-PCR**. *Vet. Immunol. Immunopathol.* 2009, **130**:114–119.

97. Zhong Q, Zhang Q, Wang Z, Qi J, Chen Y, Li S, Sun Y, Li C, Lan X: **Expression profiling and validation of potential reference genes during Paralichthys olivaceus embryogenesis**. *Mar. Biotechnol.* 2008, **10**:310–318.
