# Supplementary material for: RefGenes: identification of reliable and condition specific reference genes for RT-qPCR data normalization
Source: BMC Genomics. 2011 Mar 21;12:156. doi: 10.1186/1471-2164-12-156 (PMC3072958; doi:10.1186/1471-2164-12-156)
Supplement: Additional file 2 — Variance of gene expression across different array types. Standard deviation of signal intensity versus mean signal intensity for all probe sets from different Affymetrix array types available in Genevestigator. [file 1471-2164-12-156-S2.PDF]

Additional file 2

Variance of gene expression across different array types

Standard deviation of signal intensity versus mean signal intensity for all probe sets from different Affymetrix array types available in Genevestigator. Arrays were normlized using MAS5. Red spots indicate probe sets that had an "absent" call in more than 90 percent of arrays. The number of arrays taken into each analysis is indicated below.

A

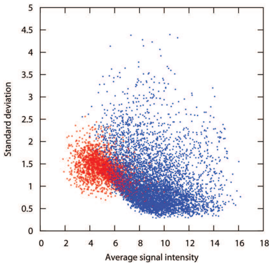

Arabidopsis AG array (8k)

B

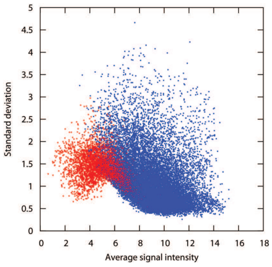

Arabidopsis ATH1 array (22k)

C

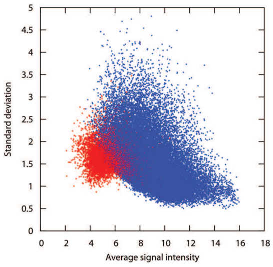

Mouse 430_2 array (40k)

D

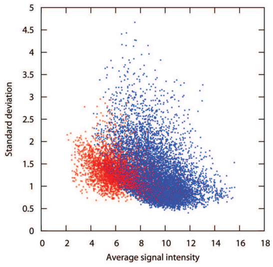

Mouse MG_U74Av2 array (12k)

E

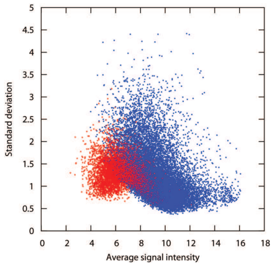

Human HG_U133A array (20k)

F

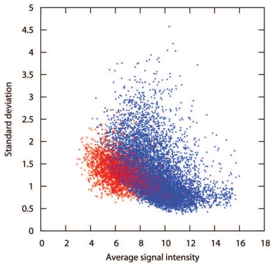

Human HG_U95A array (10k)

G

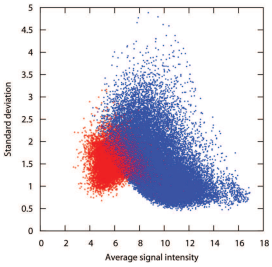

Human133_2 array (20k)

These results were obtained from datasets containing the following number of Affymetrix arrays:

A: 113	B: 4070
C: 2974	D: 3051
E: 3511	F: 1103
G: 5014	
