# Supplementary material for: RefGenes: identification of reliable and condition specific reference genes for RT-qPCR data normalization
Source: BMC Genomics. 2011 Mar 21;12:156. doi: 10.1186/1471-2164-12-156 (PMC3072958; doi:10.1186/1471-2164-12-156)
Supplement: Additional file 4 — Ranking of the SD of the most stable probe sets across different mouse tissues. Ranking of the SD of the most stable probe sets identified for a variety of mouse tissue samples (AffymetrixMouse430 2.0 platform). [file 1471-2164-12-156-S4.PDF]

Additional file 4

Ranking of the SD of the most stable probe sets identified for a variety of mouse tissue samples

The top 200 most stable probe sets for each tissue type were identified. As a control, the top 200 most stable genes across all arrays are shown.

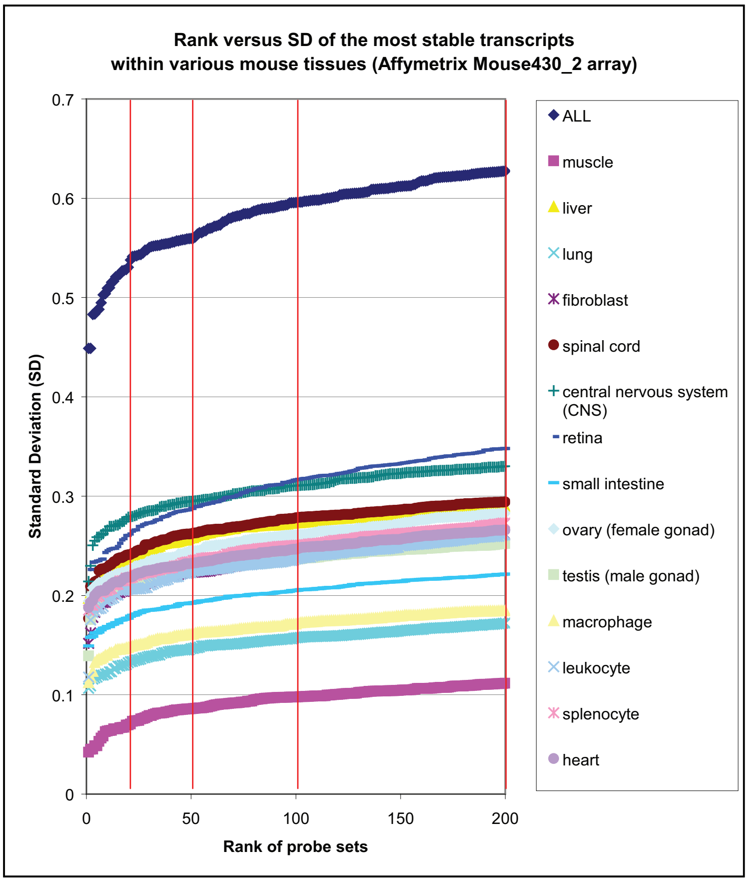

	Average ratio across all tissues
Rank 20 versus rank 1	1.32
Rank 50 versus rank 1	1.44
Rank 100 versus rank 1	1.54
Rank 200 versus rank 1	1.67
