# Supplementary material for: RefGenes: identification of reliable and condition specific reference genes for RT-qPCR data normalization
Source: BMC Genomics. 2011 Mar 21;12:156. doi: 10.1186/1471-2164-12-156 (PMC3072958; doi:10.1186/1471-2164-12-156)
Supplement: Additional file 6 — GeNorm calculations. This figure shows the complete set of GeNorm calculations for the results summarized in Figure 3 of the article. [file 1471-2164-12-156-S6.PDF]

# Additional file 6

## Complete set of GeNorm calculation iterations for each validation experiment

For each experiment, M values were calculated by GeNorm for all tested genes. In a sequential fashion, the least stable gene (i.e. the one with highest M value) was eliminated and the GeNorm algorithm was applied again to the remaining genes. This iterative process was applied until two genes were left. The ranking of the stability of gene expression was done based on the sequence of removal of the least stable genes after each iteration. The corresponding indication for expression stability was measured as the average of the M values of the remaining genes (second column).

| | Average of M values of remaining genes | Expression stability M |
|---|---|---|

| Mouse liver | | GAK | SRP72 | MRPL16 | VPS4A | ACTB | HPRT | GAPDH | TUBB |
|---|---|---|---|---|---|---|---|---|---|
| TUBB | 0.296 | 0.249 | 0.254 | 0.273 | 0.311 | 0.300 | 0.335 | 0.346 | 0.400 |
| GAPDH | 0.269 | 0.229 | 0.242 | 0.244 | 0.291 | 0.283 | 0.328 | 0.331 | |
| HPRT | 0.240 | 0.211 | 0.223 | 0.227 | 0.268 | 0.274 | 0.339 | | |
| ACTB | 0.207 | 0.200 | 0.204 | 0.196 | 0.228 | 0.251 | | | |
| VPS4A | 0.188 | 0.180 | 0.194 | 0.190 | 0.206 | | | | |
| MRPL16 | 0.171 | 0.160 | 0.182 | 0.195 | | | | | |
| SRP72 / GAK | 0.148 | 0.148 | 0.148 | | | | | | |

<::::: most stable least stable :::::>

| Arabidopsis leaves | | At3g01150 | GAPDH | At3g61710 | ACTB | At1g32050 | UBQ10 |
|---|---|---|---|---|---|---|---|
| UBQ10 | 0.631 | 0.558 | 0.529 | 0.571 | 0.777 | 0.722 | 1.042 |
| At1g32050 | 0.500 | 0.420 | 0.395 | 0.547 | 0.640 | 0.641 | |
| ACTB | 0.420 | 0.357 | 0.365 | 0.539 | 0.554 | | |
| At3g61710 | 0.306 | 0.314 | 0.297 | 0.448 | | | |
| At3g01150 / GAPDH | 0.163 | 0.163 | 0.163 | | | | |

<::::: most stable least stable :::::>

| Arabidopsis seedlings | | At3g24160 | At1g13320 | At3g27820 | GAPDH | ACTB | UBQ10 |
|---|---|---|---|---|---|---|---|
| UBQ10 | 0.323 | 0.279 | 0.314 | 0.291 | 0.348 | 0.384 | 0.432 |
| ACTB | 0.280 | 0.243 | 0.271 | 0.272 | 0.333 | 0.361 | |
| GAPDH | 0.245 | 0.256 | 0.237 | 0.241 | 0.276 | | |
| At3g27820 | 0.219 | 0.218 | 0.221 | 0.250 | | | |
| At3g24160 / At1g13320 | 0.189 | 0.189 | 0.189 | | | | |

<::::: most stable least stable :::::>

| Arabidopsis apex | | At2g17390 | At3g17920 | At5g51880 | ACTB | GAPDH | UBQ10 |
|---|---|---|---|---|---|---|---|
| UBQ10 | 0.485 | 0.458 | 0.474 | 0.504 | 0.480 | 0.510 | 1.504 |
| GAPDH | 0.223 | 0.182 | 0.205 | 0.247 | 0.257 | 0.260 | |
| ACTB | 0.197 | 0.171 | 0.208 | 0.211 | 0.253 | | |
| At5g51880 | 0.154 | 0.137 | 0.170 | 0.199 | | | |
| At2g17390 / At3g17920 | 0.108 | 0.108 | 0.108 | | | | |

<::::: most stable least stable :::::>

| Human LCLs | | EIF4EBP2 | INTS4 | SDHA | GAPD | YWHAZ | B2M | ZNF410 | BUD13 | GOLT1B | RPL13 | SAP130 |
|---|---|---|---|---|---|---|---|---|---|---|---|---|
| SAP130 | 0.231 | 0.240 | 0.244 | 0.210 | 0.235 | 0.186 | 0.195 | 0.220 | 0.238 | 0.263 | 0.278 | 0.296 |
| RPL13A | 0.220 | 0.246 | 0.250 | 0.217 | 0.239 | 0.175 | 0.182 | 0.202 | 0.224 | 0.242 | 0.258 | |
| GOLT1B | 0.210 | 0.229 | 0.232 | 0.203 | 0.226 | 0.169 | 0.179 | 0.207 | 0.233 | 0.256 | | |
| BUD13 | 0.197 | 0.210 | 0.213 | 0.186 | 0.208 | 0.167 | 0.178 | 0.221 | 0.242 | | | |
| ZNF410 | 0.183 | 0.190 | 0.193 | 0.174 | 0.193 | 0.169 | 0.177 | 0.234 | | | | |
| B2M | 0.169 | 0.167 | 0.171 | 0.156 | 0.173 | 0.180 | 0.187 | | | | | |
| YWHAZ | 0.156 | 0.150 | 0.156 | 0.149 | 0.169 | 0.201 | | | | | | |
| GAPD | 0.137 | 0.129 | 0.140 | 0.142 | 0.153 | | | | | | | |
| SDHA | 0.116 | 0.113 | 0.118 | 0.155 | | | | | | | | |
| INTS4 / EIF4EBP2 | 0.076 | 0.076 | 0.076 | | | | | | | | | |

<::::: most stable least stable :::::>

| Cattle liver | | VPS4A | GAK | ACTB | PMPCA | UBQ | GAPDH | RAB21 |
|---|---|---|---|---|---|---|---|---|
| RAB21 | 0.368 | 0.319 | 0.329 | 0.363 | 0.375 | 0.403 | 0.416 | 0.421 |
| GAPDH | 0.349 | 0.307 | 0.319 | 0.354 | 0.373 | 0.392 | 0.396 | |
| UBQ | 0.325 | 0.300 | 0.312 | 0.330 | 0.357 | 0.390 | | |
| PMPCA | 0.294 | 0.288 | 0.287 | 0.306 | 0.331 | | | |
| ACTB | 0.269 | 0.266 | 0.271 | 0.288 | | | | |
| VPS4A / ACTB | 0.249 | 0.249 | 0.249 | | | | | |

<::::: most stable least stable :::::>

| Pig liver | | Histone H3 | UBQ | VPS4A | GAK | GAPDH | PMPCA | SRP72 |
|---|---|---|---|---|---|---|---|---|
| SRP72 | 0.400 | 0.349 | 0.394 | 0.409 | 0.403 | 0.411 | 0.437 | 0.579 |
| PMPCA | 0.357 | 0.314 | 0.352 | 0.366 | 0.372 | 0.380 | 0.405 | |
| GAPDH | 0.338 | 0.304 | 0.333 | 0.367 | 0.350 | 0.370 | | |
| GAK | 0.318 | 0.288 | 0.320 | 0.347 | 0.356 | | | |
| VPS4A | 0.297 | 0.290 | 0.303 | 0.305 | | | | |
| UBQ / Histone H3 | 0.288 | 0.288 | 0.288 | | | | | |

<::::: most stable least stable :::::>
