# Supplementary material for: RefGenes: identification of reliable and condition specific reference genes for RT-qPCR data normalization
Source: BMC Genomics. 2011 Mar 21;12:156. doi: 10.1186/1471-2164-12-156 (PMC3072958; doi:10.1186/1471-2164-12-156)
Supplement: Additional file 8 — Pre-validation of reference genes for CD4 T-lymphocytes. This figure shows screen shots of meta-profile data for candidate reference genes for CD4 T-lymphocytes. [file 1471-2164-12-156-S8.PDF]

Additional file 8

Screen shots of the pre-validation of reference genes for CD4 T-lymphocytes

Reference genes for CD4 T-lymphocytes were searched with RefGenes. We then created a selection of probe sets from the proposed list of reference genes and looked at their expression characteristics across the Meta-Profile Analysis tools.

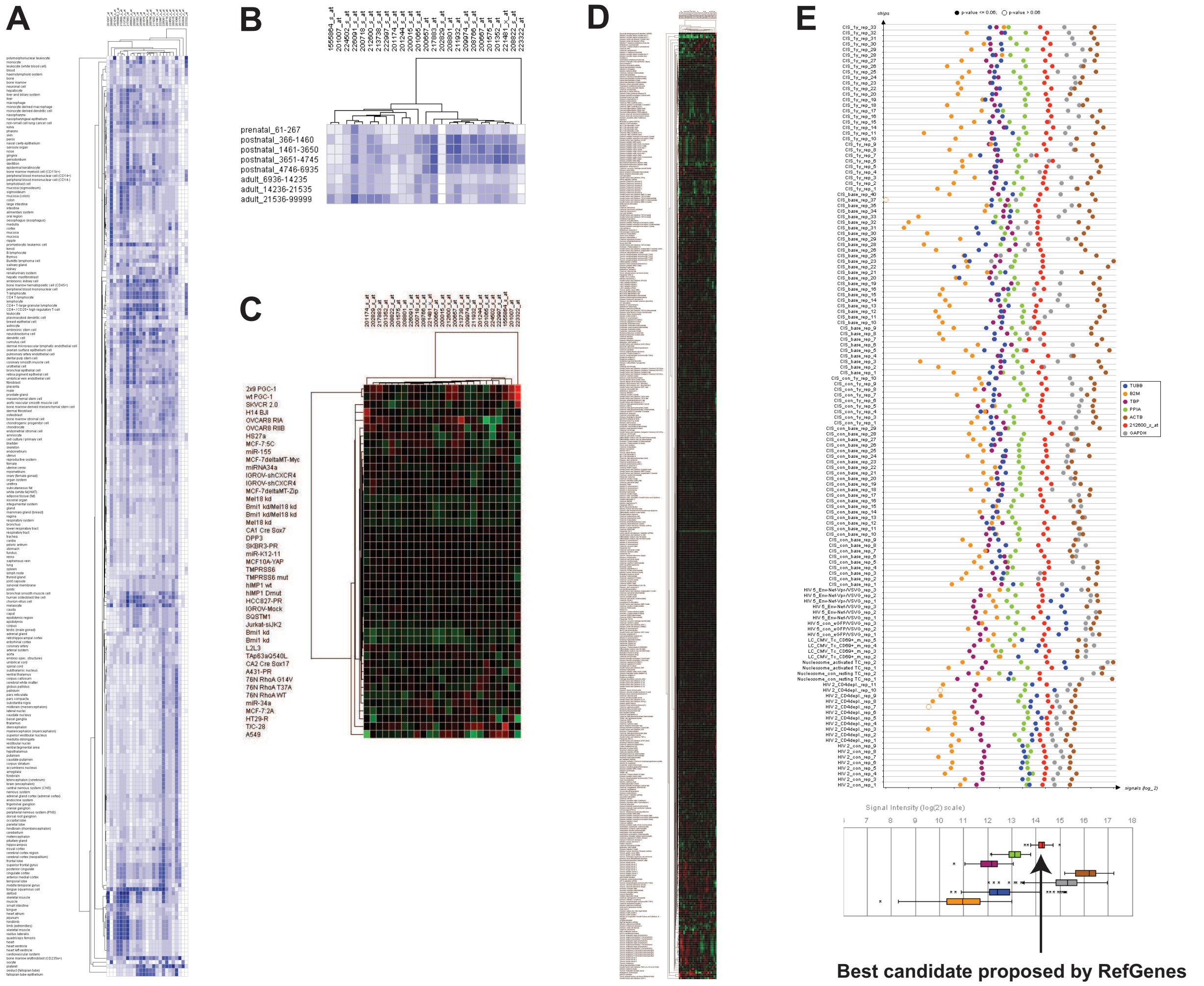

Pre-validation of new reference genes identified for CD4 T-lymphocytes (137 arrays; 5 studies) A. Hierarchical clustering of the expression profiles of these 25 gene candidates across anatomical categories. B. Expression profiles across developmental stages. C. Response to various mutated cell lines. D. Response across a large set of diseases, chemical treatments, and tumors. E. Expression characteristics of several commonly used reference genes across the 137 arrays from CD4 T-lymphocyte samples. The box and whiskers plots from RefGenes for these genes is shown, and the best novel candidate reference gene identified by RefGenes is shown in red and indicated with an arrow.
